# Feline Leishmaniasis Caused by *Leishmania infantum*: Parasite Sequencing, Seropositivity, and Clinical Characterization in an Endemic Area From Brazil

**DOI:** 10.3389/fvets.2021.734916

**Published:** 2021-08-25

**Authors:** Nara Santos dos Santos, Flaviane Alves de Pinho, Nicole Regina Capacchi Hlavac, Talyta Lins Nunes, Nádia Rossi Almeida, Manuela Silva Solcà, Bruno Milen Varjão, Ricardo Wagner Portela, Jeronimo Nunes Rugani, Felipe Dutra Rêgo, Stella Maria Barrouin-Melo, Rodrigo Pedro Soares

**Affiliations:** ^1^Laboratory of Veterinary Infectious Diseases, Teaching Hospital of Veterinary Medicine, Federal University of Bahia (UFBA), Salvador, Brazil; ^2^Department of Veterinary Anatomy, Pathology and Clinics of the School of Veterinary Medicine and Zootechny, Federal University of Bahia, Salvador, Brazil; ^3^Department of Veterinary Preventive Medicine and Animal Production of the School of Veterinary Medicine and Zootechny, Federal University of Bahia, Salvador, Brazil; ^4^Laboratory of Immunology and Molecular Biology, Institute of Health Sciences, Federal University of Bahia, Salvador, BA, Brazil; ^5^Instituto Rene Rachou, Fundação Oswaldo Cruz, Belo Horizonte, Brazil

**Keywords:** bone marrow cytology, cats, leishmaniasis, one health, PCR, zoonosis

## Abstract

Zoonotic leishmaniasis caused by *Leishmania infantum* is a disease of One Health concern since human and animal cases and environmental damage are interconnected. *L. infantum* has a complex epidemiological cycle with multiple hosts, including mammals—humans, domestic, and wild animals—and arthropod vectors. Knowledge on mammal infections in endemic areas is crucial for developing control strategies. This work aimed to detect and characterize *L. infantum* infection in domestic cats from areas where human and canine leishmaniasis cases occur. No cases of feline leishmaniasis (FeL) had been previously reported in those areas. Five municipalities from Bahia state were chosen, comprising 2,480.8 km^2^ with 1,103,866 inhabitants. Ninety domiciliated and/or sheltered cats underwent clinical examination and serology by a rapid reference test recommended by the Brazilian government. Cytology, PCR, and parasite DNA sequencing were performed in bone marrow samples. Rapid tests detected antibodies in 5.6% (5/90) of the cats. *Leishmania infantum* infection was confirmed in 7.8% (7/90) of the cats by PCR, sequencing, and parasite isolation. Three out of the five municipalities (60%) had infected cats, and PCR positivity varied from 6.9 to 29%. One cat was categorized as harboring active *L. infantum* infection with amastigote forms in bone marrow smears. No clinical signs were detected at the first clinical exam, but 1 month later the cat developed severe FeL. The cat isolate was grown in culture, typed and its DNA sequence was homologous to the *L. infantum* reference strain (PP75). In conclusion, cats are potential hosts and may acquire *L. infantum* in endemic areas where canine and human cases occur. For cats, the need for surveillance, differential diagnosis and clinical care is highly recommended since a fast clinical progression of FeL developed in a subclinical animal. An accurate standardized immunodiagnostic assay for FeL is warranted.

## Introduction

Leishmaniasis is a vector-borne disease affecting humans and animals caused by more than 20 species of the protozoan *Leishmania* (1). They are endemic in 92 countries, where more than one billion people are at risk of infection (2). Depending on the region, *Leishmania* is transmitted by different phlebotomine sand fly vectors (3). There is no human vaccine (4, 5) and the prevalence of leishmaniasis is closely linked to the human development index and environmental degradation (6, 7). This zoonosis remains a serious public health problem and is recognized as one of the 20 major neglected tropical diseases (8). The World Organization for Animal Health also lists leishmaniasis among the diseases that require worldwide notification (9). Visceral leishmaniasis (VL) is the most fatal form of leishmaniasis in humans caused by *Leishmania donovani*/*Leishmania infantum* (Old World) and *Leishmania chagasi* (same as *L. infantum*) in the New World (10).

In southern Europe, Africa, Asia, and America, *L. infantum* causes zoonotic leishmaniasis in humans (VL) (11–13) and in domestic dogs (CanL) (14, 15). *Leishmania infantum* infection has also been detected in wild animals (16, 17) and other domestic species including cats, horses, cattle, sheep, and goats (18–20). Natural infection and disease have been described in domestic cats to a lesser extent than in dogs (21–23). A recent meta-analysis including 36 years of cross-sectional studies from 12,635 cats in *Leishmania* endemic areas worldwide found an estimated seroprevalence of 11% and an overall PCR positivity of 10%. At least 63% of the infections were caused by *L. infantum* (24). Other studies detected 3–4% of cats with *L. infantum* in Italy, Greece, and German by serology (25, 26). Those recent studies have demonstrated that *Leishmania* is also circulating and cats and this should be taken as an alert in the regions where this parasite occurs.

In South America, the epidemiological cycle of *L. infantum* is complex and involves wild and domestic vertebrate and invertebrate hosts (27–30) as a result of social/economic inequality and environmental changes (31). CanL is a severe zoonosis because the dog is highly susceptible exhibiting elevated parasite loads and disease. Besides, it is very close to humans acting as a competent domestic reservoir (32, 33). In Brazil, seroprevalence in dogs and cats ranges from 2.4 to 32% (34) and 0.7 to 30.48%, respectively (35–37). However, the role of cats in *L. infantum* epidemiology is still in its infancy and should be more explored.

Recent investigations have focused on domestic cats' potential in *L. infantum* transmission (22, 38). Standardized serological tests for cats and a more detailed knowledge on their immune responses against *L. infantum* infection are needed (39) since coinfections may predispose cats to develop feline leishmaniasis (FeL) and other diseases (18, 40). These relevant aspects would improve the pivotal role of cats as *L. infantum* hosts in the context of One Health. As part of a wider study on leishmaniasis epidemiology, natural feline *L. infantum* infection from VL and CanL endemic areas is reported. Also, the clinical presentation and natural evolution of an infected cat is provided in detail.

## Materials and Methods

### Study Area, Animals, and Ethical Aspects

**Figure 1:**
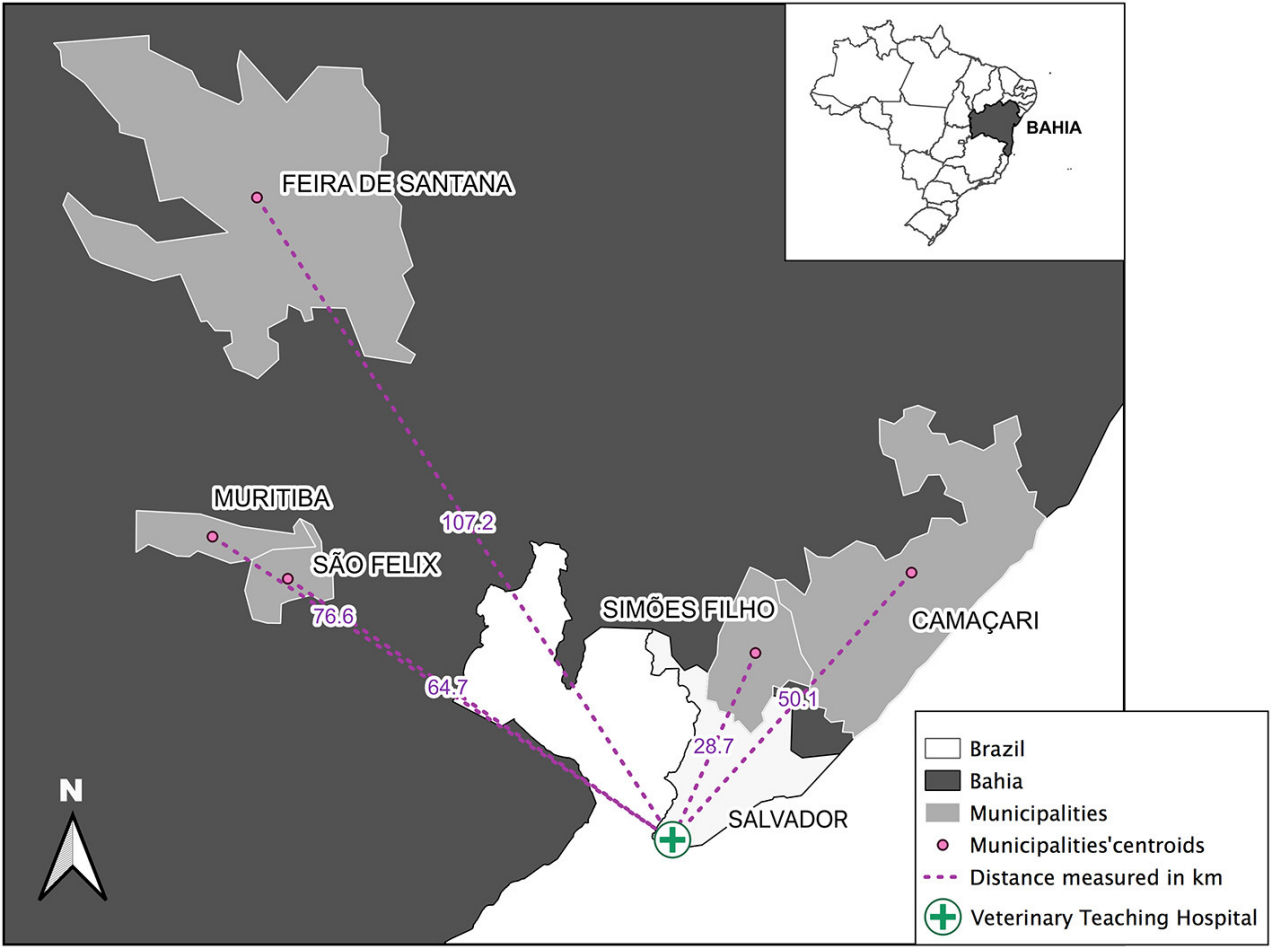
Geographical location of the surveyed municipalities (Feira de Santana, Muritiba, São Felix, Simões Filho, and Camaçari) in the State of Bahia, Brazil. Distance in kilometers (km) from each municipalities' centroids (pink dots) to the Federal University of Bahia Veterinary Teaching Hospital (green cross) were measured (pink dotted lines). Source: IBGE 2010, Datum: SIRGAS, 2000.

This study was conducted from July 2018 to December 2019 in five municipalities of Bahia state, Brazil. Those included Camaçari, Feira de Santana, Muritiba, São Félix, and Simões Filho. They are located within a 100 km radius from Salvador, the state's capital (Figure 1), comprising an area of 2,480,800 km^2^ containing a population of 1,103,866 inhabitants as estimated in 2020 (Table 1) (41). In 2010, their Human Development Index (HDI) ranged from of 0.660 to 0.712 (Table 1) (41) and, from 2009 to 2018, total human VL cases ranged from 3 to 101 (42). Feline and canine populations in the area are estimated at 2,800 to 120,000 and at 1,700 to 72,000, respectively (43).

**Table 1:** Demographic parameters and feline infection (%) by *L. infantum* by different diagnostic methods performed in 90 cats from leishmaniasis endemic regions in Bahia state, Brazil between July, 2018 and December, 2019.

**Municipalities' descriptions**						***Leishmania infantum*** **diagnostic tests** **Frequency (positive cats/tested cats)**		
**Name**	**Urban perimeter** (km^**2**^)^*^	**Estimated** population in 2020^*^	**Human development** index (HDI) in 2010^*^	**Human VL cases** (2009–2018)^*^	**CanL^**§**^** ** occurrence**	**TR-DPP^®^** **serum**	**PCR** **bone marrow**	**Cytology** **bone marrow**
São Félix	103.226	14,762	0.639	3	No record	42.9% (3/7)	29% (2/7)	14.3% (1/7)
Muritiba	86.311	29,410	0.660	7	15.7% seroprevalence; 88.8% of tested PCR^a^	3.7% (1/27)	11.1% (3/27)	0% (0/27)
Camaçari	785.421	304,302	0.694	34	21.5% seroprevalence; 70.3% positivity of tested parasite culture^b^	3.5% (1/29)	6.9% (2/29)	0% (0/29)
Simões Filho	201.418	135,783	0.675	4	No record	0% (0/17)	0% (0/17)	0% (0/17)
Feira de Santana	1,304.425	619,609	0.712	101	34.4% seropositivity frequency^c^	0% (0/10)	0% (0/10)	0% (0/10)
Total	2,480.800	1,103,866	–	149	–	5.6% (5/90)	7.8% (7/90)	1.1% (1/90)

* *Source: Brasil, 2021. Notified cases of visceral leishmaniasis (VL) caused by L. infantum from 2009 to 2018 (10 years).*

§ *Canine leishmaniasis (CanL) cases caused by L. infantum reported in the literature;*

a *Varjão et al. (44);*

b *Julião et al. (45);*

c *Deiró et al. (46)*.

In these municipalities, VL cases have been officially reported from 2009 to 2018 (42). According to the Health Ministry classification (47), Feira de Santana is the only considered an area of intense VL transmission, whereas the others are sporadic. However, CanL cases amongst the veterinary community of all municipalities is a concern.

A convenience sample of 90 cats including domiciliated (75) and sheltered (14), regardless of breed and sex, was evaluated. An active search was conducted, and 29 households and one animal shelter were included. Inclusion in the study was based on the proximity of a given CanL case either within the household/shelter or in a neighboring house. Eligibility criteria for cats in the study were: at least 6 months of age, and a signed informed consent by the owner. The exclusion criteria were: pregnancy, body weight under 2 kg and/or irascible behavior during handling.

All procedures were approved by the Committee for Ethics on the Use of Animals of the School of Veterinary Medicine and Zootechny from the Federal University of Bahia (Permission number CEUA-13/2017). The study was designed and conducted following the Brazilian Council of Animal Experimentation guidelines, and there was strict adherence to the Brazilian law for “Procedures for the Scientific Use of Animals” (No. 11.794/2008).

### Clinical Examination and Sample Collection

The cats underwent clinical evaluation including anamnesis regarding their habits and previous health conditions. Also, physical examination assessing FeL clinical signs as described by the LeishVet group was performed (48). Peripheral blood samples were collected for serology. After sedation with acepromazine (0.05 mg/kg) and meperidine (3 mg/kg), local anesthesia with lidocaine was used for bone marrow aspiration sampling at the iliac crest (49). Part of the bone marrow aspirates was used for cytological preparation; the rest was stored in RNAse and DNAse free microtubes at −20°C prior to DNA extraction. Cats with enlarged superficial lymph nodes and/or skin lesions underwent sampling by fine-needle aspiration.

### Cytological Examination

Smears of fine-needle aspiration of bone marrow, lymph nodes, and skin lesions were stained by the Romanowsky method (Panótico Rápido®, Laborclin, São Paulo) for optical microscopic search of *Leishmania* amastigote forms under 1,000 × magnification (Olympus, CX22LED).

### Serology

Because there are no standardized serological tests for cats, the reaction of anti-*Leishmania* antibodies was assessed using the canine Dual-Path Platform immunochromatographic test (TR-DPP® Biomanguinhos, Fiocruz, BR). It is a reference assay recommended by the Brazilian Ministry of Agriculture for the serodiagnosis of CanL having a sensitivity and specificity of 86 and 94%, respectively (50). It detects the reaction of total IgG against fixed recombinant *L. infantum* k28 protein as antigen using staphylococcal A protein linked to colloidal gold as conjugate (51). Protein A is also capable of binding to cat IgGs (52). A previous study reported the use of the TR-DPP® test for the serodiagnosis of *Leishmania* infection in feline sera samples (53). Reactions were conducted according to the manufacturer's instructions. We interpreted TR-DPP® positive results as an exposure to *L. infantum* but not as markers of active infection or disease.

### Molecular Procedures

Total DNA from bone marrow samples and parasite cultures were extracted using Wizard® Genomic DNA Purification Kit (Promega®, USA), according to the manufacturer's recommendations. Bone marrow DNA samples were used to amplify the endogenous control glyceraldehyde-3-phosphate dehydrogenase (GAPDH) (54) and *L. infantum* kinetoplast DNA with the RV1 and RV2 primers (55). Amplified fragments were observed in a 2% agarose gel stained with ethidium bromide (0.5 μg/mL). RV1-RV2 amplicons were sequenced and the phylogenetic trees were built using MEGA X (56). To identify the isolated strains, HSP70 and ITS1 PCR-RFLP fragments were performed (57, 58). *Leishmania* reference strains for outgroup comparison included *Leishmania braziliensis* (MHOM/BR/75/M2903), *Leishmania guyanensis* (MHOM/BR/75/M4147), *Leishmania major* (MHOM/IL/81/Friedlin), *Leishmania amazonensis* (IFLA/BR/67/PH8), and *L. infantum* (MHOM/BR/74/PP75) (59).

### Data Analysis

The data obtained in this study were tabulated in Microsoft Excel 2000 to calculate variable frequencies and perform descriptive analysis. A positive criterion for FeL was the amplification of parasite DNA by PCR in bone marrow samples. Active infection in PCR positive cats was defined by the identification of amastigotes (bone marrow, lymph node or skin) during cytological examination. Maps were constructed using the QGIS v3.8 program to display studied areas where FeL cases occurred.

### Feline *L. infantum* Active Infection

One cat with an active infection detected by cytological examination during the first investigation (T_1_) was clinically followed. It was evaluated at 1 month (T_2_) and three months (T_3_) after the second evaluation totalizing 4 months. At T_2_ and T_3_, hemogram, biochemical profile, and urinalysis were performed. Serological, parasitological, and molecular diagnosis of *L. infantum* infection were performed as described above, with the addition of a conjunctival swab collected with a sterile brush for cytological examination. Parasite load was assessed at T_1_ using duplex qPCR for simultaneous detection of *L. infantum* kinetoplast DNA (kDNA) and a conserved region of the housekeeping gene 18S rRNA. The results were expressed as parasites/mL as previously reported (60). *Leishmania infantum* isolated from bone marrow aspirates was grown in NNN-Schneider and expanded in M199 medium (61, 62) prior to DNA extraction and sequencing. The cat was also tested for feline viral leukemia virus (FeLV) and feline immunodeficiency virus (FIV) by a commercial test (SNAP Combo Test, IDEXX Laboratories) at the three evaluation points.

## Results

### Cat Population, *L. infantum* Infection, and Sequencing

All 90 samples from bone marrow were analyzed by PCR and 7.8% (7/90) were positive for *L. infantum* DNA. Those cats were then diagnosed as infected. The serological test TR-DPP® detected anti-*Leishmania* antibodies in the sera of 5.6% (5/90) of the cats. However, *L. infantum* infection was not identified by cytological and/or molecular techniques in four of them. The frequency of PCR positive results within municipalities ranged from zero to 29%. Table 1 shows the results of diagnostic tests with different types of examined biological samples. In the cat population, 77.8% (70/90) were domiciliated, 52% (47/90) males, 86.7% (77/90) adults, 96.7% (87/90) were mixed breed, and 65.6% (59/90) exhibited at least one clinical sign of disease. Table 2 shows the phenotypic characteristics and health status during physical examination of the 90 cats per locality.

**Table 2:** Parameters of the feline population in the five surveyed municipalities for *L. infantum* in the state of Bahia, Brazil (July, 2018 to December, 2019).

**Variables**	**Municipalities**					
	**Muritiba**	**São félix**	**Simões filho**	**Camaçari**	**Feira de Santana**	**Total *n* (%)**
**Age**
≥6–11 months	22.2% (6/27)	14.3% (1/7)	5.9% (1/17)	0% (0/29)	0% (0/10)	8.9% (8/90)
≥1– ≤ 8 years	74.1% (20/27)	85.7% (6/7)	94.1% (16/17)	100% (29/29)	60% (6/10)	86.7% (77/90)
>8 years	3.7% (1/27)	0% (0/7)	0% (0/17)	0% (29/29)	30% (3/10)	4.4% (4/90)
**Domiciliation**
Domiciliated	88.9% (24/27)	100% (7/7)	29.4% (5/17)	82.8% (24/29)	100% (10/10)	77.8% (70/90)
Street access	11.1% (3/27)	0% (0/7)	70.6% (12/17)	17.2% (5/29)	0% (0/10)	22.2% (20/90)
Shelter	0% (0/27)	0% (0/7)	0% (0/17)	48.2% (14/29)	0% (0/10)	15.5% (14/90)
**Sex**
Male	63% (17/27)	42.9% (3/7)	47.1% (8/17)	44.8% (13/29)	60% (6/10)	52.2% (47/90)
Female	37% (10/27)	57.1% (4/7)	52.9% (9/17)	55.2% (16/29)	40% (4/10)	47.8% (43/90)
**Breed**
Mixed breed	96.3% (26/27)	100% (7/7)	100% (17/17)	100% (29/29)	80% (8/10)	96.7% (87/90)
Persian	3.7% (1/27)	0% (0/7)	0% (0/17)	0% (0/29)	10% (1/10)	2.2% (2/90)
Siamese	0% (0/27)	0% (0/7)	0% (0/17)	0% (0/29)	10% (1/10)	1.1% (1/90)
**Physical examination**
No alteration	48.1% (13/27)	71.4% (5/7)	41.2% (7/17)	10.3% (3/29)	30% (3/10)	34.4% (31/90)
Clinical alterations	51.9% (14/27)	28.6% (2/7)	58.8% (10/17)	89.7% (26/29)	70% (7/10)	65.6% (59/90)

**Figure 2:**
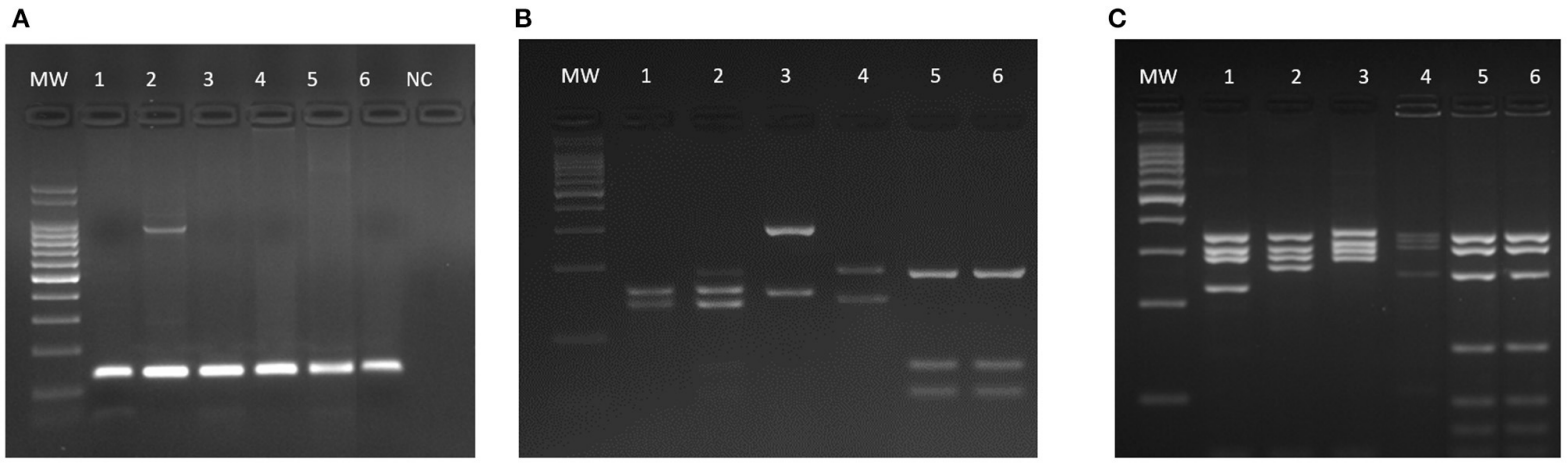
Molecular identification of *L. infantum* isolated from a cat (CAT1). Agarose gels showing the expected 145-bp fragment for RV1-RV2 PCR **(A)** and PCR-RFLP restriction profiles of ITS1 **(B)** and HSP70 **(C)** fragments. MW −100-bp molecular weight; 1–*L. braziliensis* (MHOM/BR/75/M2903); 2–*L. guyanensis* (MHOM/BR/75/M4147); 3–*L. major* (MHOM/IL/81/Friedlin); 4–*L. amazonensis* (IFLA/BR/67/PH8); 5–*L. infantum* (MHOM/BR/74/PP75); 6–CAT1 (isolated from cat); NC–negative control.

**Figure 3:**
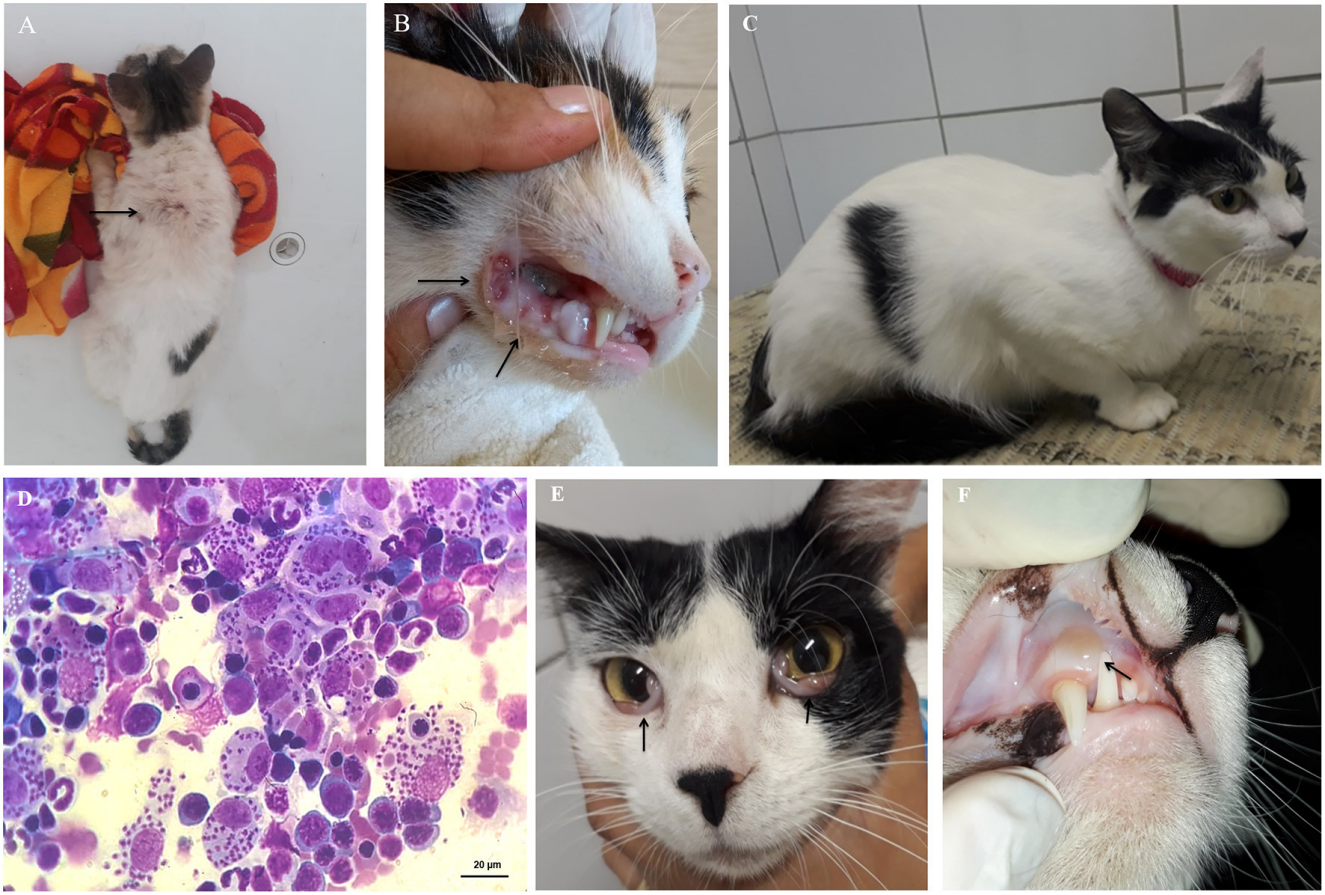
Clinical signs of feline leishmaniasis. **(A)** Areas of alopecia in the dorsal cervical region (arrow), and **(B)** ulcers in the oral cavity, suggesting gingivitis-stomatitis complex (arrows). **(C)** First physical exam of a cat with active infection without clinical signs but presenting *Leishmania* detection in bone marrow aspirates by cytological examination **(D)**. After 4 months, this cat had **(E)** bilateral oedema and hyperaemia of the conjunctiva (arrows) and **(F)** aphthous stomatitis in the upper region of the right canine tooth (arrow).

One of the PCR and DPP positive cats had *Leishmania* amastigote forms in its bone marrow aspirate (**Figure 3D**). This cat was the only categorized as having *L. infantum* active infection. There were no amastigote forms in the cytological examinations (bone marrow, lymph nodes and skin) of the remaining 89 cats. By using DNA extracted from bone marrow samples, PCR was performed using RV1–RV2 primers (Figure 2A). All positive samples exhibited the expected 145-bp fragment. After sequencing and phylogenetic analysis, those products clustered together with the *L. infantum* control (strain PP75). To confirm the taxonomic status of the *Leishmania* species infecting the only cat that we could isolated the parasite (CAT 1), two additional PCR-RFLP were performed (ITS1 and HSP70) (Figures 2B,C). Confirming the previous phylogenetic tree, the isolated strain (**lane 6**) exhibited the same restriction profile as the *L. infantum* reference strain PP75 (**lane 5**).

### Clinical Characterization of the Studied Cats

Three out of the seven cats with confirmed *L. infantum* infection by PCR had at least one suggestive clinical sign of FeL during physical examination. These alterations included weight loss, dehydration, crusted lesions on the tips of the ears, and/or areas of alopecia (Figure 3A) in the dorsal cervical region, sneezing, enlarged popliteal lymph nodes, gingivitis, and ulcers in the oral cavity suggesting gingivitis-stomatitis complex (Figure 3B).

Clinical assessment (anamnesis and physical examination) of the 83 cats diagnosed as not infected by *Leishmania—*since they were not positive either in the TR-DPP® or in the PCR test—resulted in 26.5% (22/83) of the cats with no alteration, and 73.5% (61/83) with variable clinical alterations. Signs of disease included cutaneous changes (33/83, 39.8%) characterized by focal alopecia, ulcers, crusts, abrasions, and furfuraceous dermatosis, dehydration (22/83, 26.5%), or weight loss (20/83, 24.0%) among others less frequent.

Clinical and laboratory findings in the female, mixed-breed, neutered 3-year-old pet cat described as having developed an active infection are displayed in the Supplementary Table 1, representing data from evaluations performed at three times, namely T_1_, T_2_, and T_3_. The first evaluation (T_1_) was carried out in the field. The cat was retrospectively diagnosed as having subclinical infection (PCR positivity but no clinical signs) (Figure 3C). Because there were numerous visible amastigote forms of *Leishmania* in the cytological examination of the bone marrow sample (Figure 3D), and a high parasitic load was found in the qPCR (7.7 ×10^8^ parasites/mL), the cat was referred to a thorough clinical diagnosis and the owner was instructed to keep a sandfly repellent collar in the cat. One month after the field evaluation, the cat was brought to the clinical examination (T_2_) exhibiting signs of dehydration, lymph node enlargement and a skin ulcer. Cytological preparations of bone marrow and conjunctival samples were positive for amastigote forms, and serum biochemistry showed increased total serum proteins [7.9 g/dL; reference range (RR) = 5.4 to 7.8 g/dL] and gamma-glutamyl transferase (GGT) (6.3 U/L; RR = 1.5 to 5.3 U/L) (63, 64) at T_2_. Although the cat was referred for treatment of FeL, the owner did not return until 3 months later (T_3_). At T_3_, the cat exhibited bilateral oedema and hyperaemia of the conjunctiva (Figure 3E), presence of aphthous stomatitis (Figure 3F), and enlarged lymph nodes. Hemogram evidenced lymphocytosis and serum biochemistry, hyperproteinaemia associated with hyperglobulinemia and increased serum alkaline phosphatase. At T_3_, *Leishmania* amastigotes were found in the cytological examination of multiple samples such as lymph nodes, palpebral conjunctiva, and bone marrow. Serological test TR-DPP® was performed during the three evaluations but resulted positive only for the serum sample collected at T_2_. The cat was also negative for FIV and FeLV retroviruses as tested by the lateral flow assay (SNAP Combo Test, IDEXX Laboratories) in serum samples collected at T_1_, T_2_, and T_3_.

## Discussion

This is the first study to report the infection by *L. infantum* in cats in the state of Bahia, Brazil, where 7.8% of cats were infected by *L. infantum*. The finding of *L. infantum* DNA by PCR in the bone marrow of the tested cats indicating parasite circulation simultaneously with CanL and VL that have been notified in the last 12 years (44, 65, 66). Comparatively, in other endemic Brazilian regions for both VL and CanL with similar socioeconomic and environmental characteristics, the frequency of *L. infantum* infection in cats ranged from 4.0 to 31.9% suggesting that it my vary depending on the region (21, 37, 67, 68).

Brazilian health authorities declared one of the studied municipalities (Feira de Santana) as an area of intense VL transmission, and an average of 20.2 human cases have been reported from 2014 to 2018 (42). Endemicity of zoonotic leishmaniasis in that municipality had been assessed in a former study reporting 0.6 to 2.1% of “a global prevalence of canine cases” between 1995 and 2000 (69). A recent study on CanL serology reported a seroprevalence of 34.4% in the same area (46). Although this municipality has favourable characteristics for harbouring FeL, a low adherence from the households to participate in the study was noticed. Only one pet-owner agreed to participate, and his cat was negative. This might be a result of the better knowledge of the control measures used for positive dogs. Since this is an intense VL/CanL transmission area, probably the households were aware of Brazilian health authorities' control measures. This would favour underreporting and hinder conclusions in a particular area.

There were positive cats in municipalities considered sporadic VL transmission including São Félix, Muritiba, and Camaçari (Table 1). In Muritiba, a recent study reported on a seroprevalence of 15.7% of dogs with CanL (44) whereas in Camaçari, where 11 VL cases were notified, CanL seroprevalence was 19.8% (70). In São Félix and Simões Filho, where <3 VL cases between 2014 and 2018 were notified, no available data on CanL was found. Despite gaps in notification by the health authorities, there is strong evidence of endemic zoonotic circulation of the parasite in these municipalities where local veterinarians have been increasingly reporting CanL. Consistent with these observations, the highest frequency of *L. infantum* infection found in the present study was in one of these localities (São Félix). Perhaps the lack of positivity in two of the municipalities investigated in this study was because of the low number of animals and households evaluated.

PCR was chosen as a confirmatory method of infection due to its high sensitivity and ability to confirm parasite species (71, 72). Among the 90 examined cats from a highly endemic area, the frequency found of *L. infantum* positivity was 7.9%. Moreover, only one out of seven infected cats developed FeL, suggesting that they may not be as susceptible to infection as dogs. In this context, other studies suggested that felines appear to be resistant to infection by *L. infantum* (73, 74). A lower tissue parasitism in cats compared to dogs might hinder cytological examination less accurate during diagnosis. It has been demonstrated in previous studies on dogs that the spleen is the best choice for a more sensitive parasitological or molecular detection of *L. infantum* (14, 71, 75). Moreover, studies have showed that a higher parasitic load in the canine host is associated with more intense disease, and that these more susceptible dogs are more infectious to the phlebotomine vectors (33, 76). Factors such as the parasite's tissue tropism and intrinsic pathophysiological mechanisms make organs such as the spleen more intensely parasitised than other ones in infected dogs (77–79). Thus, in dogs, the use of samples from such organs is likely to improve diagnostic sensitivity of methods based on the detection of parasites and/or its DNA (71, 75, 80). All these aspects have crucial importance under the view of One Health and remain to be more investigated in cats in the diverse endemic regions for *L. infantum* infection not only in Brazil, but also in other countries. Here, we advocate that feline bone marrow could be one of the target organs for FeL since we detected a higher parasite load in infected macrophages (Figure 3C). Bone marrow is commonly a chosen target for sampling tissue for PCR diagnosis because it is known to be consistently parasitised in dogs (71) and humans (81) with *Leishmania* active infection. Bone marrow has been shown to be a suitable sample for the diagnosis of *L. infantum* infection in cats by PCR (73, 82). Bone marrow collection under proper sedation also causes a minor impact on the welfare of cats (49). Consistent with those observations, the present study reports the identification of *L. infantum* in different cats by using several PCR techniques and sequencing.

Five cats tested positive in TR DPP®, but no parasite DNA was detected in the bone marrow in four of them. These data may suggest a natural lesser susceptibility in cats whose residual antibody response might only indicate a transient parasite exposure. Besides, cross-reactions with other pathogens should be considered, as already reported for dogs (83, 84). Susceptible canine hosts develop high antibody levels as the infection progresses, together with the development of clinical alterations and a higher chance to infect vectors (66, 79, 85, 86). Based on our data, this does not seem to happen in cats. Furthermore, the TR-DPP® used in the present study might not have detected feline immunoglobulins against *L. infantum* which would certainly exist as a consequence of the constant exposure to infected vectors (87). Indeed, among the seven cats that were positive for *L. infantum* DNA detection in the PCR test, only one was seropositive in the TR DPP®. Moreover, the only cat with active infection described herein had a negative result in the TR DPP® assay at the very moment when it was exhibiting overt signs of severe FeL. We emphasise that we have evaluated cats that were highly exposed to infection because of their close coexistence with infected dogs in areas of high transmission. Comparatively, a study on *L. infantum* naturally infected cats reported a considerable 75%-seropositivity for anti-*Leishmania* immunoglobulins as detected by an in-house indirect ELISA test (22). Thus, the lateral flow assay used in this study does not seem to be accurate for detecting feline antibodies against *Leishmania* antigens. This reinforces the need of more studies on immune responses during FeL and the development of more specific tests for *L. infantum* detection in cats.

Typically, because of the PCR's high sensitivity to detect *L. infantum* DNA, a positive result can be obtained with minimum amounts of parasites from a host sample (88). In this sense, a positive PCR result may indicate an initial infection or even an infection under the host immune system's resolution process. To characterize an active infection that may progress to the clinical disease, cytological examinations were performed on different tissue samples from the seven PCR positive cats, and of the remaining four cats that were positive only in the lateral flow assay. The molecular approaches confirmed the isolated strain to be *L. infantum*. This reinforces that several diagnostic methods should be employed for a correct diagnosis not only in dogs but especially in cats when a specific method warrants further development.

Three *L. infantum*-infected (PCR-positive) cats showed clinical signs ranging from cutaneous to systemic alterations suggestive of FeL as previously described (18). However, there was no laboratory evidence of active infection as established in our diagnosis criteria. One of these cats exhibited gum lesions indicative of a gingivitis-stomatitis complex. This cat lived in a shelter in close contact with other cats and dogs, some with diverse diseases. Living in an overpopulated environment is a well-described stress factor for cats. This favours immunosuppression and susceptibility to pathogens and other non-infectious morbidities (89). Likewise, clinical signs observed in the other two infected cats might have been associated with other pathogens since they had no *Leishmania* in their cytological samples and their condition improved upon further examinations and symptomatic treatment.

Among the substantial majority (92.2%) of PCR negative cats for *L. infantum* infection, a high proportion (73.5%) exhibited clinical signs of disease that could be attributed to FeL in a presumptive approach (18). We emphasise that the region is endemic for many other infectious diseases, and that these cats are subjected to the same conditions as their owners, namely undernourishment, high demographic densities, and precarious sanitation infrastructure. Therefore, the clinical presentation of unhealthy cats living in endemic areas for *L. infantum* does not necessarily reflect an active infection by this parasite. Accordingly, a careful assessment linking PCR positivity to clinical signs must be pursued during the clinical evaluation of a presumptive feline case. Indeed, in a different context of coinfections, a recent study performed in an endemic region for CanL in Spain, on a population of clinically healthy stray cats, still found a 5.6% positivity in blood samples using qPCR (90).

The clinical course of the cat with an active infection in this study suggests a natural pathophysiological evolution in a susceptible mammal host (91, 92). In the first examination, the cat had no clinical alteration, but its bone marrow was already heavily infected. No proper clinical follow-up and treatment could be performed because of the owner's non-compliance. Therefore, after the cat develop FeL, the owner looked for assistance again. Then, we could describe the natural infection progression. Some studies relate FeL onset to immunosuppressive factors such as retroviral infections, such as FeLV or FIV (18, 40). There was no evidence of a pre-existing infectious immunosuppressive condition in the cat with FeL. In the fourth month after the first PCR diagnosis, clinical signs consistent with FeL were also apparent in different bodily systems, confirming the disease's systemic character. Lesions in tissues such as ocular conjunctiva, oral mucosa, in addition to enlargement of lymph nodes, as observed in the present cat, have also been previously described by other authors (18, 93, 94). Hyperproteinaemia associated with hyperglobulinemia was the most evident clinical pathological finding when the cat exhibited overt clinical FeL. This finding agrees with other authors that considered hyperglobulinemia as the most relevant alteration found in FeL (95). Importantly, the proportions of gamma globulins in cats with FeL have been described as variable and often not detectable in serological tests, probably due to a predominantly cellular immune response (18, 96, 97).

The cat identified with active FeL herein lived in close contact with a dog previously diagnosed with CanL, and this dog could have been a parasite source for vectors. Indeed, a recent study on sheltered dogs and cats showed that dogs could be more infectious to sandflies due to higher parasite loads than cats with the same PCR-positivity rates. Moreover, the study argued that cats were more frequently seropositive than dogs (22). Despite xenodiagnoses techniques already proving the transmission of the parasite in sand fly feeding on infected cats, no consistent data has demonstrated that *L. infantum*-infected cats can maintain the parasite circulation in the environment (48, 98, 99). Besides, there is no robust data estimating the transmission risk for humans who cohabit with infected felines (38).

Brazilian municipalities with the highest number of VL reports low human development indexes (7, 100). In this context, as expected, cats of low-income individuals do not receive proper health care. They may be more susceptible to developing active infections with high parasite loads and therefore possibly contributing to the parasite transmission. As we mentioned before, the current study has some limitations especially due to underreporting in some areas. The studied feline population sample that could be assessed did not allow a precise prevalence estimation of the parasite infection. In the locality with the highest notification numbers of VL, a low number of cats could be evaluated, and these animals resided in the same household, so there is a possibility that the negative results found might not reflect reality. Yet, we can assume that TR-DPP® was not a reliable method for assessing humoral responses *L. infantum*-infected cats. Nevertheless, despite all limitations, the present study confirmed that *L. infantum* is circulating among cats using parasitological and molecular methods.

Since the evolution of the infection in cats is often subclinical and with a low parasitic burden, more detailed studies with representative samples are needed to ascertain *L. infantum* prevalence in endemic areas (73, 94, 101). Different from dogs, cats exhibit a more nocturnal behaviour and may walk longer distances and environments. This may increase the chances to encounter a sand fly in nature and acquire and/or transmit the parasite. More importantly, co-infections with other feline endemic pathogens including viruses (FIV, FeLV) (102), bacteria (*Mycoplasma haemofelis, Ehrlichia* spp) (103, 104) other protozoans (*Cytauxzoon* spp.) (105), and, helminths (enteric or lung worms) (106) may lead to immunosuppression of the cats. They would be more susceptible to *L. infantum* infection thus favouring higher parasite burdens. Indeed, in a recent study in Italy and Greece, where *L. infantum* may also occurs in cats and dogs, *Aelurostrongylus abstrusus* and *Troglostrongylus brevior* were found in cats and in a fatal case in a dog (107–109). Those worms have been increasingly reported as important in feline medicine and co-infections would impact animal management. Thus, further investigations should attempt to characterize hosts and co-infections in the epidemiological cycle of zoonotic leishmaniasis. This would promote better control and prevention measures to achieve the principles of One Health.

## Conclusions

Molecular identification and sequencing of *L. infantum* from naturally infected cats indicates that the parasite is circulating in areas where CanL and VL are endemic. These data demonstrate the feline's exposure to the parasite and reinforce the need for further epidemiological investigations to understand how cats participate in the cycle of this zoonotic leishmaniasis. Additionally, the clinical evolution of a subclinical infection in a cat toward the development of patent feline leishmaniasis (FeL) illustrates a picture of active infection. A specific immunodiagnostic assay to assess feline humoral response to *L. infantum* is needed.

## Data Availability Statement

The original contributions presented in the study are included in the article/Supplementary Material, further inquiries can be directed to the corresponding author/s.

## Ethics Statement

The animal study was reviewed and approved by Committee for Ethics on the Use of Animals of the School of Veterinary Medicine and Zootechny of the Federal University of Bahia (Permission number CEUA-13/2017). Written informed consent was obtained from the owners for the participation of their animals in this study.

## Author Contributions

SB-M and FP conceived and designed the study. NS, BV, TN, JR, and FR performed the investigation and drafted the manuscript. NH, NA, and RP provided laboratory material resources and validation of experiments. NS, FP, NH, NA, RP, MS, SB-M, and RS analyzed the data. FP and MS conducted statistical analyses, data curation, and visualization. SB-M, FP, and RS provided funding acquisition, project management, experiments supervision, and manuscript editing. All authors contributed to the writing of the manuscript and approved the submitted version.

## Conflict of Interest

The authors declare that the research was conducted in the absence of any commercial or financial relationships that could be construed as a potential conflict of interest.

## Publisher's Note

All claims expressed in this article are solely those of the authors and do not necessarily represent those of their affiliated organizations, or those of the publisher, the editors and the reviewers. Any product that may be evaluated in this article, or claim that may be made by its manufacturer, is not guaranteed or endorsed by the publisher.
