# Supplementary material for: Feline Leishmaniasis Caused by Leishmania infantum: Parasite Sequencing, Seropositivity, and Clinical Characterization in an Endemic Area From Brazil
Source: Front Vet Sci. 2021 Aug 25;8:734916. doi: 10.3389/fvets.2021.734916 (PMC8424124; doi:10.3389/fvets.2021.734916)
Supplement: Supplementary file 1 [file Table_1.docx]

**Supplementary Table 1.** Clinical pathology results of a cat with active infection caused by *L. infantum* at two evaluation time points after the first examination, from the Recôncavo region, Bahia state, Brazil

| **Parameter** | **Reference range*** | **T_2_^a^** | **T_3_^b^** |
| --- | --- | --- | --- |
| **Hematological** | | | |
| Red blood cells (x10³/μL) | 5.0-10.0 | 6.51 | 7.02 |
| Hemoglobin (g/dL) | 8.0 – 15.0 | 8.3 | 8.5 |
| Hematocrit (%) | 24 – 45 | 26.6 | 27.9 |
| VGM (fL) | 39.0 – 55.0 | 41.0 | 39.8 |
| CHGM (%) | 30 – 36 | 31.2 | 30.4 |
| Leukocytes (mm^3^) | 5.500 – 19.500 | 6.900 | 17.300 |
| Band neutrophil (mm^3^) | 0 – 300 | 0 | 0 |
| Segmented neutrophil (mm^3^) | 2.500 – 12.500 | 2.553 | 6.920 |
| Lymphocytes (mm^3^) | 1.500 – 7.000 | 4.278 | 9.515 (**↑**) |
| Monocytes (mm^3^) | 0 – 800 | 69 | 173 |
| Eosinophils (mm^3^) | 0 – 1.500 | 69 | 692 |
| Platelets (x10^3^/mm^3^) | 230 – 680 | 324 | 191.000** |
| Total proteins (g/dL) | 6.0 – 8.0 | 8.4 (**↑**) | 8.4 (**↑**) |
| **Biochemical** | | | |
| Total protein (g/dL) | 5.4 – 7.8 | 7.9 (**↑**) | 8.8 (**↑**) |
| Albumin (g/dL) | 2.1 - 3.3 | 3.0 | 2.9 |
| Globulin (g/dL) | 2.6 - 5.1 | 4.9 | 5.9 (**↑**) |
| Urea (g/dL) | 42.8 - 64.2 | 46.0 | 53.0 |
| Creatinine (g/dL) | 0.8 - 1.8 | 1.5 | 1.3 |
| ALT (U/L) | 6 – 83 | 28.0 | 34.0 |
| Alkaline phosphatase (U/L) | 25 – 93 | 65.0 | 109.0 (**↑**) |
| GGT (U/L) | 1.5 - 5.3 | 6.3 (**↑**) | 3.5 |

*Reference range (Jerry Kaneko et al., 2008; Weiss and Wardrop 2010). ** Platelet count may be underestimated due to the presence of platelet aggregates. (↓) Values below the normal range for the species. (↑) Values above the normal range for the species. ^a^ T_2_ = clinical examination and samples collected at 30 days after T_1_ (T1 was the day of the first examination, which intended only to collect samples for serology and *Leishmania infantum* DNA and parasite detection by PCR and cytology). ^b^ T_3_ = clinical examination and samples collected at 90 days after T_2_.
